# Chemical Characterization and Quantification of Silver Nanoparticles (Ag-NPs) and Dissolved Ag in Seafood by Single Particle ICP-MS: Assessment of Dietary Exposure

**DOI:** 10.3390/ijerph18084076

**Published:** 2021-04-13

**Authors:** Alfina Grasso, Margherita Ferrante, Giovanni Arena, Rossella Salemi, Pietro Zuccarello, Maria Fiore, Chiara Copat

**Affiliations:** 1 Department of Medical, Surgical and Advanced Technologies “G.F. Ingrassia”, University of Catania, Via Santa Sofia 87, 95123 Catania, Italy; agrasso@unict.it (A.G.); pietro.zuccarello@unict.it (P.Z.); mfiore@unict.it (M.F.); ccopat@unict.it (C.C.); 2 Freelance Chemist, 96011 Augusta, Italy; giovanniare@yhaoo.it; 3 Department of Biomedical and Biotechnological Sciences, University of Catania, Via Santa Sofia 83, 95123 Catania, Italy; rossellasalemi2580@gmail.com

**Keywords:** Ag, nanoparticle, spICP-MS, processed food, seafood, dietary intake

## Abstract

This study provides a first insight on the chemical characterization and quantification of silver nanoparticles (AgNPs) and dissolved Ag in processed canned seafood products, where food-grade edible silver (E174) is not intentionally added nor is the nanoparticle contained in the food contact material. The aim was to evaluate the bioaccumulation potential of AgNPs and to contribute to the assessment of AgNPs and ionic Ag human dietary intake from processed seafood. It is known how seafood, and in particular pelagic fish, is a precious nutritional source of unsaturated fatty acids, protein, and different micronutrients. Nevertheless, it may cause possible health problems due to the intake of toxic compounds coming from environmental pollution. Among emerging contaminants, AgNPs are widely applied in several fields such as biomedicine, pharmaceutical, food industry, health care, drug-gene delivery, environmental study, water treatments, and many others, although its primary application is in accordance with its antimicrobial property. As a consequence, AgNPs are discharged into the aquatic environment, where the colloidal stability of these NPs is altered by chemical and physical environmental parameters. Its toxicity was demonstrated in *in-vitro* and *in-vivo* studies, although some findings are controversial because toxicity depends by several factors such as size, concentration, chemical composition, surface charge, Ag^+^ ions released, and hydrophobicity. The new emerging technique called single-particle inductively coupled plasma mass spectrometry (spICP-MS) was applied, which allows the determination of nanoparticle number-based concentration and size distribution, as well as the dissolved element. Our findings highlighted comparable mean sizes across all species analysed, although AgNPs concentrations partly follow a trophic level-dependent trend. The low mean size detected could be of human health concern, since, smaller is the diameter higher is the toxicity. Dietary intake from a meal calculated for adults and children seems to be very low. Although seafood consumption represents only a small part of the human total diet, our findings represent a first important step to understand the AgNPs dietary exposure of the human population. Further studies are needed to characterize and quantify AgNPs in a large number of food items, both processing and not, and where AgNPs are added at the industrial level. They will provide a realistic exposure assessment, useful to understand if AgNPs toxicity levels observed in literature are close to those estimable through food consumption and implement data useful for risk assessors in developing AgNPs provisional tolerable daily intake.

## 1. Introduction

The last decade has been characterized by a growing concern in the research field of nanoparticles (NPs) for their several applications, because of their new and better properties dependent on size, surface area, distribution and morphology.

In particular, silver nanoparticles (AgNPs) are widely applied for more than 244 consumer products [1] in several fields such as biomedicine, pharmaceutical, food industry, health care, drug-gene delivery, environmental study, water treatments, and many others, although its primary application is in accordance with its antimicrobial propriety [2,3,4,5,6,7].

In the food industry, AgNPs have a wide spectrum of action in favor of the greatest safety and longest shelf life of food [8]. Ag is authorized as a food additive in its elemental form (E174) to be used to color the external coating of confectionery, for decoration of chocolates and in liqueurs according to the Regulation (EC) No. 1129/2011 and specification to the Regulation (EC) No. 231/2012. Nevertheless, according to the EFSA Panel on Food Additives and Nutrient Sources added to Food [9], several pieces of information need to be addressed to deep scientific knowledge on E174 particle size distribution, the release of Ag ions from elemental silver, and data on toxicity studies on elemental silver. Furthermore, AgNPs is widely used in food-contact plastics in order to provide an antimicrobial activity to the food product as well improve its properties [10], being the efficiency of AgNPs much stronger compared to the bulk Ag such as Ag-binding zeolites, based on the surface area to volume ratio [11]. Currently, AgNPs are not included among the authorized substances given in Commission Regulation (EU) No. 10/2011. Accordingly, a maximum level of 0.01 mg/kg in food should be established for the migration of a non-authorized substance through a functional barrier.

As a consequence of their numerous uses, AgNPs are discharged into the aquatic environment, where the colloidal stability of this NP is altered by chemical and physical environmental parameters [12], thus influencing AgNPs dissolution to give Ag^+^ when the temperature increase or the pH decrease [13], or depending by the available natural organic matter (NOM) [14]. The dispersion of AgNPs into the natural environment could lead to the bioaccumulation process and determine trophic transfer to food webs, thus implying also risks for human health [1].

Nevertheless, in addition to the engineered metal nanoparticles (ENPs), AgNPs exists also as naturally occurring. Since natural waters contain Ag(I) ions, in the presence of natural organic matter such as humic acids [15], AgNPs are formed under thermal, non-thermal, and photochemical conditions [16]. Furthermore, they can be released by erosion, geological weathering, and other phenomena such as fires [17].

Consequently, humans could be exposed to NPs every day through food, water, air, and dermal contact. From the literature is known that silver in the human body does not play any biological role and the majority is removed, while 1–2% is accumulated in the organism [18].

The toxicity of AgNPs has been studied in the laboratory with concentrations typically higher (10 mg/L) than those found in the natural environment and concerning also several form and size [1]. The AgNPs act decreasing the antioxidant enzymes, imbalance the oxidative status, altering the mitochondrial membrane potential, inducing cell death, DNA damage, and cytokines secretion [19,20,21,22]. On the other hand, it is poorly understood if the toxicity of AgNPs can be attributable to its particulate form combined with the ionic silver released, since NPs facilitates more rapid dissolution of ions than the equivalent bulk material [23]. Accordingly, results from the literature are controversial, suggesting that both ionic and particulate Ag should be taken into consideration in the toxicological evaluation of AgNPs [24].

With this study, we wanted to provide a chemical characterization and quantification of AgNPs and dissolved Ag, in processed canned seafood products belonging to different trophic positions with the emerging technique of Single Particle—Inductively Coupled Plasma (*sp*ICP-MS), which allows the determination of particle number-based concentration, with rapid simultaneous characterization of its elemental composition, number of particles, size and size distribution, in addition to the dissolved concentration. Our choice to carry out this study using canned seafood was because it is a product that makes up a significant contribution to the diet (unsaturated fatty acids, protein, and different micronutrients), nevertheless, it may cause possible health problems due to the intake of toxic compounds. Furthermore, we also provided data related to the estimated daily intake for both adults and children, which may be useful to risk assessors, to develop a provisional tolerable daily intake for AgNPs.

## 2. Materials and Method

### 2.1. Sample Collection, Handling and Preparation

The research material consisted of canned seafood products belonging to three different fish species and one mollusc species, purchased in the period between June and July 2019, from Italian supermarkets chains in the city of Catania (Italy). Different brands for canned tuna (5), mackerel (4), anchovy (3), and clam (3) were chosen among the more popular ones and three different batches have been selected for each brand. The extraction and analysis procedure on each sample was carried on in triplicate, obtaining 45 extractions for canned tuna, 36 for canned mackerel, 27 for canned anchovy, and 27 for canned clam. The content of every can was homogenized and next the samples were frozen at −80 °C until analysis.

### 2.2. Alkaline Digestion and spICP-MS Analysis of Silver Nanoparticles (AgNPs) and Dissolved Silver (Ag)

The method described by Gray et al. [25], was used to allow an alkaline digestion of the samples. Briefly, 0.25 gr of the wet sample was weighed directly in graduated polypropylene tubes (DigiTUBEs, SCP Science, Baie D’Urfé, QC, Canada) using an analytical balance, and mixed with 5 mL of tetramethylammonium hydroxide (TMAH, 20% *v*/*v*). Firstly, a vortex was used to facilitate the separation of tissues from the walls of containers used for digestion.

The extraction was obtained through sonication for 30 min at 37 °C by an ultrasonic bath, which allows the destruction of tissues and to release of nanoparticles without altering them. Next, the samples were left to digest another 24 h at room temperature. Finally, the digested solutions were diluted to 50 mL using high purity water 0.22µm filtered (Millipore, Bedford, MA, USA) to 1% TMAH concentration and 0.1% Triton X-100, useful to prevent particle aggregation.

AgNPs and dissolved silver were analyzed with the Syngistix Nano application software supported by the ICP-MS (NexION^®^ 350D, Perkin Elmer, Waltham, MA, USA), which is gaining increasing popularity for the characterization of particles concentration and size distribution at a short dwell time, in addition to the dissolved concentration. The instrumental condition for the determination AgNPs and dissolved Ag are reported in Table 1.

All digested samples and calibration solutions were sonicated for 30 min before analysis to maximize a homogeneous dispersion. Calibration standards for analysis of dissolved Ag were prepared from a single standard solution (1000 mg/L, CPAchem, Roma, Italy).

The transport efficiency was determined using a certified reference material (AgNPs 39 ± 5 nm, 6.1 × 10^10^ particles/mL, monitoring *m/z* 107), obtaining values in the range 1.8–4.6%; while for the preparation of calibration standards was used AgNPs of 80 nm (AgNPs 80 ± 9 nm, 7.4 × 10^9^ particles/mL). Both AgNPs reference materials (PELCO^®^ 40 nm and 80 nm, Citrate, NanoXact^TM^, Ted Pella Inc., Redding, CA, USA) used were purchased from Nanovision (Brugherio, MB, Italy).

Firstly, it was evaluated an analytical recovery by spiking 40 nm Ag standard to ultrapure water, at a concentration of 20 µg/L or 5.7 × 10^7^ particles/mL, and obtaining a median diameter around 41.8 ± 3.5 nm and a recovery of 94.1% (5.4 × 10^7^ ± 3.1 × 10^6^ particles/mL), following the manufacturer’s declaration.

Furthermore, it was studied the effect of the extracting solution on the particle’s concentration and size distribution, spiking the AgNPs standard in both ultrapure water (n_1_ = 7) and TMAH 1% (n_2_ = 7), at the same concentration of 150 ng/L or 4.5 particles/mL × 10^5^.

The results obtained were very similar and statistically homogeneous if applied a two tailed t-test (*p* = 95%; ν = n_1_ + n_2_ − 2 = 14), and showing no effect from the extraction solution on the nanoparticles concentration or on size distribution (mean size: t_calculated_ = 0.69 < t_tabulated_ = 2.14; particles/mL: t_calculated_ = 1.67 < t_tabulated_ = 2.14): 4.2 × 10^5^ ± 4.9 × 10^4^ particles/mL and 42.1± 3.3 nm of mean size in water and 3.8 × 10^5^ ± 3.6 × 10^4^ particles/mL and 43.1 ± 1.90 nm of mean size in TMAH 1%, with a recovery of 97.0% and 88.4% respectively.

The limit of detection (LOD) and the limit of quantification (LOQ) were calculated by analyzing ten alkaline extract blanks, in the same analytical condition of the samples, and based respectively on the mean ± 3 SD and the mean ± 10 SD criterion of the number of particles/mL obtained. LOD was 1.5 × 10^3^ particles/mL, while LOQ was 3.0 × 10^3^ particles/mL. Referring to the sample weight and digestion volume used, they resulted in 3.3 × 10^5^ particles/g and 5.9 × 10^5^ particles/g, respectively.

Besides, LOD in size (LOD nm) was estimated at 20 nm applying the following Equation (1) [26,27]:

(1) $$\mathrm{LODnm}=\sqrt[{3}]{\frac{6\times 3\sigma_{blank}}{R\times f_{a}\times \rho \times \pi }}\;$$

where: $3\sigma_{blank}$ is three times the standard deviation of counts/dwell time of alkaline blanks (1% TMAH); R is the slope of the calibration curve of ionic Ag solutions; f_a_ is the mass fraction of analyzed metallic element in the AgNPs; ρ is the density of the AgNPs.

Accuracy has been assessed spiking silver NPs 40 nm, at a concentration of 20 µg/L or 5.7 × 10^7^ particles/mL, in seafood samples, one for each batch of analysis, obtaining a mean particles recovery of 88.5 ± 4.2%.

As can be inferred from the results obtained, the recovery of AgNPs particles in extracting solution is significantly lower than those in ultrapure water, probably depending by an agglomeration phenomenon.

For this reason, we performed also the evaluation of total silver, after acid digestion, intending to confirm the characterization of AgNPs using spICP-MS analysis.

### 2.3. Acid Digestion and ICP-MS Analysis of Total Silver (Ag)

To confirm the efficiency of alkaline digestion on the extraction of AgNPs, an assisted microwave acid digestion was performed for the evaluation of total silver.

About 0.5 gr of the wet sample were weighed directly in Teflon vessels and then 6 mL of 67% super pure nitric acid (HNO_3_, Carlo Erba, Milano, Italy) and 2 mL of 30% hydrogen peroxide (H_2_O_2_, Carlo Erba) were added to each sample. A blank sample containing only the reagents was prepared for every mineralization batch.

The microwave mineralization was performed stepwise up to 200 °C in 10 min (1000 W), followed by a 15 min rest at 200 °C (1000 W).

After, the cooled samples digested were transferred into graduated polypropylene tubes and diluted to 50 mL using high purity deionized water. Before analysis, all samples were filtrated through 0.45 µm nylon filters, pre-washed with 5 mL 10% *v*/*v* HNO_3,_ and rinsed with 5 mL ultrapure water.

For the determination of total silver an inductively coupled plasma mass spectrometer was used (ICP-MS NexION^®^ 350D, Perkin Elmer), and the instrumental conditions are reported in Table 2.

ICP-MS quantification of total Ag was carried out, in the standard mode, using the standard addition technique, covering a concentration range from 1 to 10 µg/L, using the same single standard solution (1000 mg/L, CPAchem). Yttrium (Y89) was selected as the internal standard.

The analytical process was controlled using the measurement of Laboratory Fortified Matrix (LFM) with a seafood sample (spike of ionic silver at 20 µg/L) processed at each batch of digestion. The recoveries calculated are all in the range 94–117%.

The LOD and LOQ were calculated by analyzing ten acid extract blanks based on the mean ± 3 SD/mean ± 10 SD criterion. They resulted in 0.012 and 0.025 mg/kg, respectively.

### 2.4. Dietary Exposure

The Estimated Meal Intake (EMI) (µg/Kg b.w. per day) derived from the consumption of selected seafood products was conducted for AgNPs and dissolved Ag according to the following equation [28]:

EMI = (C × M)/BW(2)

where C is the AgNPs or dissolved Ag (mg/Kg w.w.); M is the meal size (227 g for adults and 114 g for child); BW is the body weight, considered as 16 Kg for child (6 years) and 70 Kg for adult (70 years) [29,30].

To evaluate if the intake of dissolved Ag derived from seafood consumption could represent a risk for a human to develop chronic systemic effects, we estimated the Target Hazard Quotient (THQ). When THQ reports a value below 1 means that the level of exposure is smaller than the oral reference dose (RfD), otherwise a daily exposure at this level is not likely to cause any deleterious effects during their exposure duration for the human population. THQ is calculated according to the following equation [31]:

THQ = (EF × ED × IR × C)/(RfD × BW × AT)(3)

where EF is the exposure frequency or the number of exposure events per year of exposure (350 days/year); ED is the exposure duration (adults 26 years; children 6 years); IR is the average consumption of seafood for the Italian population (applied as 12.16 g/capita/day for pelagic fish CT, CM, and CA; 12.34 for molluscs CC); C is the metal concentration in the food (mg/Kg w.w.); RfD is the oral reference dose (0.005 mg/Kg day) [32]; BW is the body weight (the same used for EDI); and AT is the averaging time (equal to EF × ED).

### 2.5. Determination of Packaging Composition

To verify the percent presence of Ag in packaging composition, fragments of them were analyzed by scanning electron microscopy coupled with microanalysis using a Stereoscan 360 instrument (Cambridge Instruments, Cambridge, UK) combined with an X energy dispersion detector (SEM-EDX) Miniflex diffractometer (Rigaku, Austin, TX, USA) having the Inca software. For each brand and for each species of canned product, four representative points were scanned for a qualitative analysis, taking into account the internal intact layer, two breaking points, and the external intact layer.

### 2.6. Statistical Analysis

The statistical software package IBM SPSS 20.0 (IBM, Armonk, NY, USA) was used for statistical analysis. Differences in AgNPs and dissolved Ag levels among selected seafood products were studied, and one-way analysis of variance (ANOVA) coupled with a post hoc Tukey test were applied.

## 3. Results

**Figure 1:**
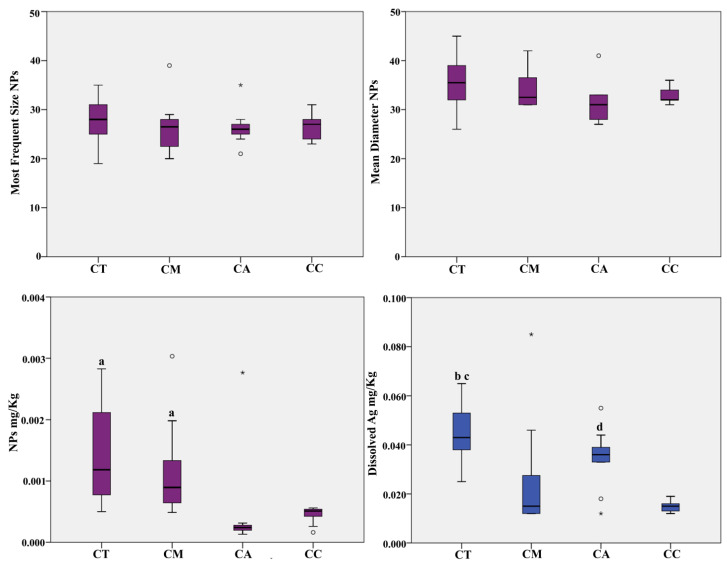
Box Plot distribution of AgNPs most frequent size (nm), mean diameter (nm), level of NPs, and dissolved Ag (mg/Kg) in packaged seafood products. Legend: CT, Canned Tuna; CM, Canned Mackerel; CA, Canned Anchovy; CC, Canned Clam; a, *p* < 0.05 vs. CA and CC; b, *p* < 0.01 vs. CM; c, *p* < 0.001 vs. CC; d, *p* < 0.05 vs. CC. ° Outliers values of the distribution. * Extreme values of the distribution.

The AgNPs, as well as the level of Ag in its dissolved form, were analysed with the Syngistix Nano Application software supported by the ICP-MS NexION^®^ 350D instrument (Perkin Elmer), by processing the samples with the ultrasound-assisted alkaline digestion. The total Ag was measured with ICP-MS measurements after acid digestion of the samples and, as shown in Table 3, there are no significant differences between dissolved fraction and total Ag, highlighting the efficiency of a properly NPs extraction during the first experimental procedure. As shown in Table 3 and Figure 1, selected seafood products did not show differences regarding the most frequent size and mean diameter. Only two samples of canned mackerel have the most frequent size below LOD (20 nm). The highest most frequent size was measured for another sample of canned mackerel (39 nm) and, the mean of most frequent size is in the range of 26–28 nm for all the seafood products analysed. Regarding the mean diameter, it was measured a size range of 31–36 nm among selected seafood products, with values progressively higher according to the following order: canned anchovy < canned clam < canned mackerel < canned tuna.

As regards the AgNPs level, in term of number of AgNPs/g and AgNPs mg/Kg, the samples of canned tuna and canned mackerel had the higher levels than canned anchovy and canned clam, with a mean value of 2.28 × 10^7^ and 1.86 × 10^7^ number of AgNPs/g respectively, which correspond to a concentration of 0.0014 and 0.0012 mg/kg respectively. In canned clam and canned anchovy, these concentrations were in the range of 0.44 × 10^7^–0.91 × 10^7^ number of AgNPs/g, which correspond to a concentration of 0.0004 and 0.0005 mg/kg. Although all the reported values seem to be very low, none of the measured samples had a concentration lower than LOD (3.3 × 10^5^ number of AgNPs/g; 3.4 × 10^−5^ mg/Kg).

Dissolved Ag was found with concentration progressively higher with the following order: canned clam < canned mackerel < canned anchovy < canned tuna. Canned tuna and canned anchovy did not report significant differences in concentration, with values of 0.0346 and 0.0455 mg/Kg respectively. Nevertheless, values measured in canned tuna are significantly higher than canned mackerel (0.0245 mg/Kg) and canned clam (0.0148 mg/Kg), whereas concentrations measured in canned anchovy were significantly higher only versus canned calm. For few values were measures concentrations below LOD (0.012 mg/Kg). These were reported for 4 samples of canned mackerel, 2 samples of canned clam, and 1 sample of canned anchovy.

**Figure 2:**
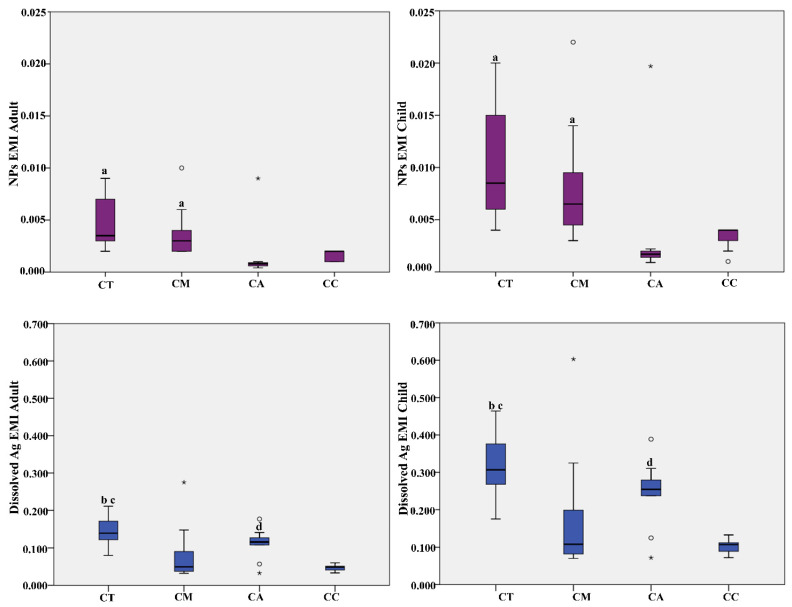
Box Plot distribution of Estimated Meal Intake (EMI µg/Kg b.w.) of AgNPs and dissolved Ag concentration calculated for adult (70 years) and child (6 years). Legend: CT. Canned Tuna; CM, Canned Mackerel; CA, Canned Anchovy; CC, Canned Clam; a. *p* < 0.05 vs. CA and CC; b *p* < 0.01 vs. CM; c *p* < 0.001 vs. CC; d *p* < 0.05 vs. CC. ° Outliers values of the distribution. * Extreme values of the distribution.

In Table 4 and Figure 2 are shown results regarding the Estimated Meal Intake (EMI) of AgNPs and dissolved Ag, calculated both for adult (70 years old) and child (6 years old), deriving from consumption of the selected seafood products. Since exposure dose reflects both concentrations measured in seafood products, body weight, and meal ingestion rate, EMI is significantly higher in the child than in adults, both for AgNPs and dissolved Ag.

As regard AgNPs, in both age classes, EMI is significantly higher if consuming canned tuna and canned mackerel than canned anchovy and canned clam. We obtained EDI mean values of 0.0047 and 0.0102 µg/Kg b.w. deriving from a meal of canned tuna, respectively for adult and child, and EDI mean values of 0.0037 and 0.0082 µg/Kg b.w., deriving from a meal of canned mackerel, respectively for adult and child.

Concerning dissolved Ag, EMI is significantly higher if consuming canned tuna and canned anchovy than canned mackerel and canned clam. We obtained EMI mean values of 0.1474 and 0.3238 µg/Kg b.w. deriving from a meal of canned tuna, respectively for adult and child, and EMI mean values of 0.1108 and 0.2435 µg/Kg b.w. deriving from a meal of canned anchovy, respectively for adult and child. These values are below the oral daily reference dose set for inorganic Ag (5 µg/Kg b.w.) and, THQ results below 1 for both adults and children, if an exposure scenario of 6 and 26 years of duration is chosen for the two age groups respectively.

**Figure 3:**
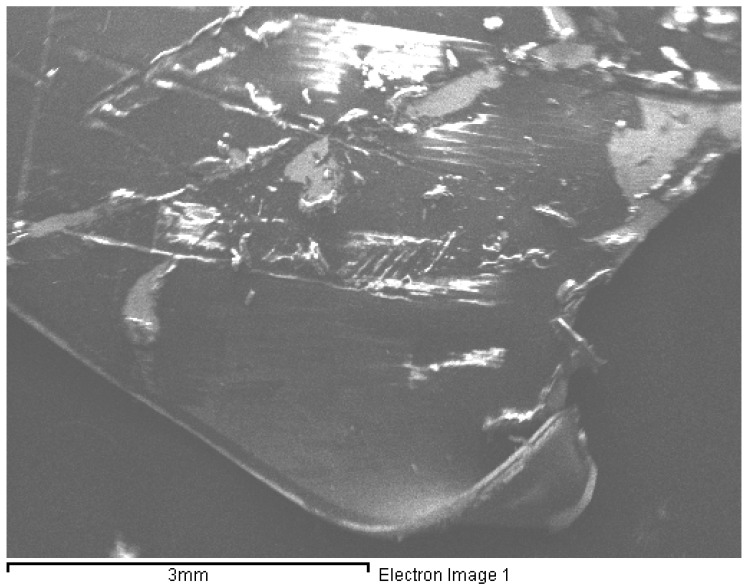
Example of a box fragment scanned by a Stereoscan 360” combined with an X Energy Dispersion Detector (SEM-EDX).

The SEM analysis on packaging did not show the presence of Ag in any of the scanned points. Microanalysis mainly highlighted, in addition to the presence of oxygen (O), the presence of iron (Fe), tin (Sn) and zinc (Zn) (Table 5, Figure 3). On the inner surface of the packaging, that is the one in contact with food, the predominant presence of carbon (C) makes the use of organic coatings, such as epoxy resins, likely.

## 4. Discussion

This study provides for the first time the characterization and quantification of AgNPs, as well as the quantification of dissolved Ag, in processed canned seafood belonging to the best-selling brands available in large supermarket chains. The additive E174, based on Ag in its elemental form, was not intentionally added in none of the chosen products. As well, since the packages of all the selected canned products were made of metal, they do not fall within active packages obtained by the addition of nano compounds with antimicrobial properties, such as AgNPs [8]. Moreover, the SEM analysis performed in our study did not show the presence of Ag in their composition. For the above reasons, we supposed that a bioaccumulation process of Ag as nanoparticles and/or ionic form was occurred in the natural environment of seafood and/or during a contamination in the food processing at the industrial level.

Once discharged in the marine environment, the fate, distribution, and environmental impact of AgNPs is poorly understood yet. Nevertheless, it seems that environmental matrices influence particle dissolution and aggregation [33], undergoing to a chemical modification such as adsorption of organic molecules or reaction with dissolved species [34]. When AgNPs are dispersed in a saline environment, and thus with high ionic strength, the aggregation is favoured [35]. Conversely, the high chloride content in seawater paired with the natural organic matter (NOM), favours AgNPs dissolution [34]. Otherwise, most AgNPs remain suspended when a low ionic strength occurs, representing a potentially negative threat to the biota in an ionic or nanoscale form, while in a more saline environment nanoparticles agglomerate and precipitate on the surface of the sediment [36]. The results obtained from Xu et al. [37] on seawater analyses showed a particle concentration (0.54 × 10^7^ particles/mL) and a size distribution of AgNPs (30.5 nm) similar to those obtained in our research, suggesting the possibility of environmental contamination. Nevertheless, because in seawater chlorides precipitate as AgCl(s), and if in excess they form the dissolved ion AgCl^2-^, most of the intake may have occurred in the seafood under this chemical form. Subsequently, in the gastric tract characterized by an acidic pH, Ag^+^ can be released, and when enters the basic intestinal environment or the blood circulation, the AgNPs could be formed again. Bioaccumulation of total Ag is not extensively studied in seafood of marine environment as other metals and metalloids [37,38,39,40,41], and the available concentrations from the literature are highly variable. For instance, in coastal sharks from The Bahamas, Ag in muscle tissue was found at a concentration between 0.055 and 0.518 mg/Kg w.w., with no accumulation trend associated with size [42]. In Indian mackerel captured along the coastal waters of Visakhapatnam, India, Ag in muscle tissue was found with concentrations ranging from 0.10 to 1.20 mg/Kg w.w. [43]. A bioaccumulation study conducted in commercial marine fish from Brazil revealed Ag concentrations in muscle tissue ranging from 0.055 to 0.166 mg/Kg d.w. [44]. Trace levels of Ag were also found in the muscle of fish from the Gulf of Lions (North-West Mediterranean Sea) and the Bay of Biscay (North-East Atlantic Ocean), with a concentration between 0.008 and 0.026 mg/Kg d.w. [45]. In canned fish of 6 different fish species were found a mean concentration of 0.0053 mg/Kg w.w. [18]. In freshwater environments, the main fishery from the La Plata basin (South America) revealed muscle levels of Ag below the limit of detection [46]. Similar results were reported for freshwater fish from the New Caledonia lagoon [47], in fish collected from a fish farm located in Nahuel Huapi National Park in the northern Patagonia Andes Mountains, Argentina [48] and in fish collected from a fish farm from Beijing [49]. Irrelevant levels of Ag bioaccumulation described for freshwater ecosystems can be accountable to a low bioavailability of Ag or to the impact of low salinity on Ag bioaccumulation potential. A study conducted along Tunis lagoon, revealed high bioaccumulation concentration in clams sampled during the warmest seasons (0.34–0.60 mg/Kg d.w.) compared to the coldest ones (<LOD—0.41 mg/Kg d.w.), the latest associated with low water salinity [50].

The concentration of Ag we found in processed fish falls within the range provided by the literature for the marine ecosystem, and results do not show a trend associated with the trophic level, being Ag of canned tuna > canned anchovy > canned mackerel. To the best of our knowledge, our study is the first to deal with the characterization and quantification of AgNPs in seafood. As it is described in the results section, the mean size of AgNPs (31.1–35.8 nm), as well the most frequent size (26.2–27.7 nm), are comparable for all fish studied and, they did not show a trend associated with the trophic level, as it was observed for TiO_2_NPs [28]. Conversely, the number of AgNPs/g is significantly higher in fish with a larger size, tuna, and mackerel, than anchovy, as we partially reported also for TiO_2_NPs [28]. Accordingly, if an accumulation of NPs will be verified in humans, being at the top of the food chain, we are potentially exposed to higher concentration than those found in nature.

Only one study in literature reports concentration and characterization of both AgNPs and Total Ag in marine bivalve collected from an offshore aquaculture farm. Clam samples belonging to the species *Ruditapes philippinarum* revealed an Ag concentration of 0.90 mg/Kg w.w., a mean size of AgNPs of 44.8 nm, and an AgNPs/g number of 2.07 × 10^7^ [37]. Compared to our study, the results we reported are lower for both Ag (0.015 mg/Kg w.w.), number of AgNPs/g (0.44 × 10^7^), and mean size (32.9 nm). Furthermore, the mean size of AgNPs in the canned clam is in the same diameter range found for fish species, and the number of AgNPs/g is the lowest value among all canned seafood analysed. However, despite the concentrations of Ag^+^ and AgNPs found in our study have been lower than ones reported in other studies, the ratio between the number of AgNPs and milligrams of dissolved Ag (very close to the total) is about 10 times greater in our results compared to those reported in the literature. Therefore, even if the total Ag amount has been lower, in our study Ag showed proportionally a greater tendency to aggregate into NPs having smaller sizes. The slight discrepancy observed between measurements can be linked to the variation of water chemistry, including ionic strength, natural organic matter, pH, and dissolved oxygen [37]. The low mean size detected could be of human health concern, since, as it was shown from literature data, smaller is the diameter higher is the toxicity following oral exposure, given the ability of nanoparticles to move better into the circulatory system [37].

Although the number of AgNPs reported for canned seafood seems very high, its corresponded concentration in terms of mg/Kg w.w., as well the estimated daily intake for adults and children, is very low. It ranges from 0.0016 to 0.0047 µg/Kg b.w. for adults and from 0.0033 to 0.0102 to 0.0047 µg/Kg b.w. for children. Conversely, the estimated daily intake of the dissolved ion fraction is significantly higher, ranging from 0.0047 to 0.147 µg/Kg b.w. for adults and from 0.102 to 0.324 µg/Kg b.w. for children. Nevertheless, these concentrations are lower than the oral Reference Dose (RfD) set for the inorganic silver (0.005 mg/Kg day b.w.) [32,51] and, according to the results of THQ, there is no risk to develop chronic systemic effects due to dissolved Ag intake during the exposure duration applied.

Scientific opinion shows a great concern regarding the risk for human and environmental health, even at low concentrations, deriving from the toxicity of AgNPs which depend on several factors such as size, concentration, chemical composition, surface charge, Ag^+^ ions released, and hydrophobicity [52,53]. That said, chemical features and behavior of AgNPs in the different exposure matrices can result in contrasting findings relating to toxicity. Literature data report *in-vitro* genotoxicity of AgNPs, for eg. in human bronchial epithelial cells HBEC3-kt, human TK6 cells, human keratinocytes (SVK14), and mouse fibroblasts (NIH3T3) [54,55,56], nevertheless, genotoxicity depend on the characteristics of NPs and the type of cells exposed [54]. Regarding Ag^+^, no genotoxicity was found nor in vitro [56] and after oral exposure in a mouse model [57]. In-vivo studies revealed how AgNPs can enter the blood circulation, cross the blood-brain barrier and bioaccumulate in different organs after oral administration [53,58,59,60]. A dose-dependent accumulation was observed in rats exposed to AgNPs, with higher bioaccumulation in females than males [58,60]. Oral exposure to AgNPs in mice showed a size-dependent accumulation of AgNPs, with higher bioaccumulated concentrations for smaller diameters of NPs, as well as adverse health effects on liver and kidney and inflammatory responses when mice were exposed for 28 days to 1.00 mg/kg of AgNPs of 42 nm [53]. High subacute toxicity and oxidative damage were also observed in mice after oral exposure to 10–250 mg/kg body weight per day for 28 days, with significant pathological changes in the lung and liver [59]. Weight loss, altered liver enzymes, altered blood biochemistry values, and some loss of renal function were also observed [52,61]. In-vivo experiments on plants (*Allium cepa* and *Nicotiana tabacum*) and animals (Swiss albino male mice) showed impairment of nuclear DNA [62]. Furthermore, it was shown that AgNPs caused genotoxicity in the bone marrow of rats [63,64] and reticulocytes of mice [22] after oral exposure. Nevertheless, particle coatings can modulate nanoparticles behavior in the gastrointestinal tract and change genotoxicity effects. It was observed in a mouse model, where citrate-coated AgNPs in digestive juices tends to agglomerate much more than polyvinylpyrrolidone (PVP)-coated AgNPs becoming more genotoxic [57]. Furthermore, once ingested, AgNPs could play a role in the dysregulation of gastrointestinal functions by altering mRNA expression that regulates intestinal permeability [65].

In-vitro and in-vivo studies suggest that the toxicity of AgNPs is dependent on the silver ion fraction in the AgNPs suspension [66,67]. Differences between oral exposure to AgNPs and ionic Ag have been studied and a greater bioaccumulation was observed for the ionic form [58,67,68]. Nevertheless, high fecal excretion following oral administration of AgNPs was observed [59,68], whereas that of ionic Ag is significantly lower [68].

Conversely, other studies did not show any adverse effects in liver and kidney, or hematological and histopathological changes in animals treated with polyvinyl pyrrolidone coated AgNPs (PVP-AgNPs) after sub-chronic oral exposure [69]. Acute oral exposure to 2000 mg/kg of AgNPs in rats did not show genotoxicity or mortality [70], whereas the LD_50_ of the AgNPs synthesized from *Elaeodendron croceumin* in Wister rats was determined to be greater than 2000 mg/kg body weight. As well, *in-vivo* one-month exposure of adult zebrafish to concentrations of AgNPs (8, 45, and 70 μg/L) did not show evident morphological and ultrastructural organization in cornea epithelium [71].

Several issues still need to be better addressed such as the capacity of AgNPs to enter the body themselves reaching the various body districts or if only the silver ions issuing from the NPs are absorbed and which chemical reactions undergo in the digestive tract based on their chemical composition [52]. Accordingly, it was observed that AgNPs capped with citrate and not, aggregate and partially react to form silver chloride during exposure to synthetic stomach fluid [72,73] and, the aggregation of nanoparticles is favoured by smaller diameters, prolonged period, as well as by the surface features of NPs [72]. Concerning the capacity of nanoparticles to interact with the biomolecules, the study of Marchiore et al. [40] investigated the migration of added silver nanoparticles from antimicrobial edible coating to sausages. They showed as silver ions, slowly released from AgNPs, are able to interact with proteins and lipids, generating a lipid oxidation and suggesting that silver may act as an activation factor in the free radical reaction.

The studies aimed to verify bioaccumulation of AgNPs after oral administration [5,53,59,60] did not quantify the specific accumulation of AgNPs in animal body district. Indeed, they analysed total Ag content with the standard technique of atomic absorption spectrometer or inductively coupled plasma. Therefore, one of the most important challenges in the field of AgNPs toxicity, or more, in general, the NPs toxicity, is a better understanding of the chemical transformations occurring once the gastrointestinal system is reached and chemical features paired with concentrations of NPs once they are translocated in tissues and organs.

## 5. Conclusions

In this study, we provided a first quantification and characterization of AgNPs in canned seafood where food-grade E174 is not intentionally added nor is the nanoparticle contained in the food contact material. Thus, it is supposed that concentrations found originate from bioaccumulation of AgNPs in the marine environment or a chemical transformation of accumulated Ag compounds once entered in organism body districts. Alternatively, the presence of AgNPs in analysed processed food could arise from contamination during food processing, at the industrial level.

Our findings highlighted comparable mean sizes across all species analysed, regardless of trophic level, although AgNPs concentrations partly follow a trophic level-dependent trend. The low mean size detected could be of human health concern, as well as the bioaccumulation potential along the food chain. Nevertheless, the concentration of AgNPs and dissolved Ag found in muscle tissue of processed seafood and the resulting EMI calculated for adults and children seems to be very low. Although seafood consumption represents only a small part of the human total diet, our findings represent a first important step to understand the AgNPs dietary exposure of the human population. Further studies are needed to characterize and quantify AgNPs in a large number of food items, both processing and not, and where AgNPs are added at the industrial level. They will provide a realistic exposure assessment, useful to understand if AgNPs toxicity levels observed in literature are close to those really estimable through food consumption and implement data useful for risk assessors in developing AgNPs provisional tolerable daily intake.

## Figures and Tables

**Table 1:** NexION^®^ 350D ICP-MS instrumental condition for single particles analysis.

Parameter	Value
Nebulizer, Flow	Meinhard, 1 mL/min
Spray chamber	Glass cyclonic
Sample uptake rate	0.26–0.28 mL/min
RF power	1600 W
Analysis mode	Standard
Quadrupole settling time	0 µs
Analyte	Ag 107
Dwell time	50 µs
Data acquisition time	60 sec
Density	10.49 g/cm^3^
Ag mass fraction	100%

**Table 2:** NexION^®^ 350D ICP-MS instrumental condition for Total Ag in standard mode.

Parameter	Value
Nebulizer, Flow	Meinhard, 0.89 mL/min
Spray chamber	Glass cyclonic
RF power	1600 W
Analogic phase voltage	−1950 V
Pulses voltage	1300 V
Discriminator threshold	12
Deflector voltage	−12 V
Analysis mode	Standard
Analyte	Ag 107
Internal standard	Y

**Table 3:** Descriptive statistics concerning the chemical characterization and quantification of AgNPs and dissolved Ag (mg/Kg) in packaged seafood products.

**Canned Tuna**	**Most Frequent Size AgNPs (nm)**	**Mean Diameter AgNPs (nm)**	**Number of AgNPs/g**	**AgNPs mg/Kg**	**Dissolved Ag ^a^ mg/kg**	**Total Ag ^b^ mg/kg**	
Mean	27.7	35.8	2.28 × 10^7^	0.0014	0.0455	0.0594	
S.D.	4.76	5.13	0.88 × 10^7^	0.0008	0.0119	0.0409	
Min.	<20	26.0	0.46 × 10^7^	0.0005	0.0251	0.0521	
Max.	35.0	45.1	3.13 × 10^7^	0.0028	0.0652	0.0781	
**Canned Mackerel**	**Most Frequent Size AgNPs (nm)**	**Mean Diameter AgNPs (nm)**	**Number of AgNPs/g**	**AgNPs mg/Kg**	**Dissolved Ag ^a^ mg/kg**	**Total Ag ^b^ mg/kg**	
Mean	26.2	34.1	1.86 × 10^7^	0.0012	0.0245	0.0374	
S.D.	5.13	3.65	0.69 × 10^7^	0.0007	0.0219	0.0316	
Min.	<20	31.2	1.18 × 10^7^	0.0005	<0.012	<0.012	
Max.	39.3	42.3	3.04 × 10^7^	0.003	0.0850	0.0990	
**Canned Anchovy**	**Most Frequent Size AgNPs (nm)**	**Mean Diameter AgNPs (nm)**	**Number of AgNPs/g**	**AgNPs mg/Kg**	**Dissolved Ag^a^ mg/kg**	**Total Ag ^b^ mg/kg**	
Mean	26.4	31.1	0.91 × 10^7^	0.0005	0.0346	0.0480	
S.D.	3.81	4.37	0.31 × 10^7^	0.0008	0.0129	0.0228	
Min.	21.2	27.4	0.49 × 10^7^	0.0001	<0.012	<0.012	
Max.	35.4	41.5	1.40 × 10^7^	0.0028	0.0550	0.0810	
**Canned Clam**	**Most Frequent Size AgNPs (nm)**	**Mean Diameter AgNPs (nm)**	**Number of AgNPs/g**	**AgNPs mg/Kg**	**Dissolved Ag^a^ mg/kg**	**Total Ag ^b^ mg/kg**	
Mean	26.6	32.9	0.44 × 10^7^	0.0004	0.0148	0.0206	
S.D.	2.72	1.76	0.17 × 10^7^	0.0001	0.0024	0.0133	
Min.	23.2	31.2	0.14 × 10^7^	0.0002	<0.012	<0.012	
Max.	31.4	36.3	0.64 × 10^7^	0.0006	0.0190	0.0321	

^a^ Ultrasound-assisted alkaline digestion and spICP-MS determination. ^b^ Microwave-assisted acid digestion and ICP-MS determination in standard mode.

**Table 4:** Descriptive statistics of Estimated Meal Intake (EMI µg/Kg b.w.) calculated for adult (70 years) and child (6 years) concerning the AgNPs and dissolved Ag and, THQ calculation concerning dissolved Ag for adult (70 years) and child (6 years).

**Canned Tuna**	**EMI Adult AgNPs**	**EMI Child AgNPs**	**EMI Adult Dissolved Ag**	**THQ Adult Dissolved Ag**	**EMI Child Dissolved Ag**	**THQ Child Dissolved Ag**
Mean	0.0047	0.0102	0.1474	1.58 × 10^−3^	0.3238	6.92 × 10^−3^
S.D.	0.0026	0.0056	0.0391	/	0.0861	/
Min.	0.0021	0.0040	0.0802	/	0.1752	/
Max.	0.0092	0.0202	0.2113	/	0.4641	/
**Canned Mackerel**	**EMI Adult AgNPs**	**EMI Child AgNPs**	**EMI Adult Dissolved Ag**	**THQ Adult Dissolved Ag**	**EMI Child Dissolved Ag**	**THQ Child Dissolved Ag**
Mean	0.0037	0.0082	0.0781	8.51 × 10^−4^	0.1713	3.72 × 10^−3^
S.D.	0.0023	0.0054	0.0717	/	0.1574	/
Min.	0.0022	0.0030	<0.038	/	<0.085	/
Max.	0.0100	0.0221	0.2752	/	0.6034	/
**Canned Anchovy**	**EMI Adult AgNPs**	**EMI Child AgNPs**	**EMI Adult Dissolved Ag**	**THQ Adult Dissolved Ag**	**EMI Child Dissolved Ag**	**THQ Child Dissolved Ag**
Mean	0.0016	0.0036	0.1108	1.20 × 10^−3^	0.2435	5.26 × 10^−3^
S.D.	0.0028	0.0061	0.0431	/	0.0946	/
Min.	0.0004	0.0009	<0.038	/	<0.085	/
Max.	0.0092	0.0197	0.1769	/	0.3887	/
**Canned Clam**	**EMI Adult AgNPs**	**EMI Child AgNPs**	**EMI Adult Dissolved Ag**	**THQ Adult Dissolved Ag**	**EMI Child Dissolved Ag**	**THQ Child Dissolved Ag**
Mean	0.0017	0.0033	0.0467	5.22 × 10^−4^	0.1024	2.28 × 10^−3^
S.D.	0.0005	0.0011	0.0084	/	0.0188	/
Min.	0.0010	0.0013	<0.038	/	<0.085	/
Max.	0.0022	0.0042	0.0601	/	0.1332	/

**Table 5:** Data about packaging microanalysis for main revealed elements: mean percentage values, standard deviation (SD), minimum (Min) and maximum (Max) values.

Descriptive Statistics	%C	%Fe	%Zn	%Sn	%O
Mean	25.1	5.18	0.10	0.40	68.9
SD	2.22	6.61	0.13	0.29	4.25
Min	22.7	0.20	0.00	0.00	64.2
Max	27.0	12.4	0.24	0.68	72.2

## Data Availability

No new data were created or analyzed in this study. Data sharing is not applicable to this article.
